# Identification and investigation of ORFans in the viral world

**DOI:** 10.1186/1471-2164-9-24

**Published:** 2008-01-19

**Authors:** Yanbin Yin, Daniel Fischer

**Affiliations:** 1 Computer Science and Engineering Dept, 201 Bell Hall, University at Buffalo, Buffalo, NY 14260-2000, USA; 2 Bioinformatics/Dept. of Computer Science, Ben Gurion University, Beer-Sheva 84015, Israel

## Abstract

**Background:**

Genome-wide studies have already shed light into the evolution and enormous diversity of the viral world. Nevertheless, one of the unresolved mysteries in comparative genomics today is the abundance of ORFans – ORFs with no detectable sequence similarity to any other ORF in the databases. Recently, studies attempting to understand the origin and functions of bacterial ORFans have been reported. Here we present a first genome-wide identification and analysis of ORFans in the viral world, with focus on bacteriophages.

**Results:**

Almost one-third of all ORFs in 1,456 complete virus genomes correspond to ORFans, a figure significantly larger than that observed in prokaryotes. Like prokaryotic ORFans, viral ORFans are shorter and have a lower GC content than non-ORFans. Nevertheless, a statistically significant lower GC content is found only on a minority of viruses. By focusing on phages, we find that 38.4% of phage ORFs have no homologs in other phages, and 30.1% have no homologs neither in the viral nor in the prokaryotic world. Phages with different host ranges have different percentages of ORFans, reflecting different sampling status and suggesting various diversities. Similarity searches of the phage ORFeome (ORFans and non-ORFans) against prokaryotic genomes shows that almost half of the phage ORFs have prokaryotic homologs, suggesting the major role that horizontal transfer plays in bacterial evolution. Surprisingly, the percentage of phage ORFans with prokaryotic homologs is only 18.7%. This suggests that phage ORFans play a lesser role in horizontal transfer to prokaryotes, but may be among the major players contributing to the vast phage diversity.

**Conclusion:**

Although the current sampling of viral genomes is extremely low, ORFans and near-ORFans are likely to continue to grow in number as more genomes are sequenced. The abundance of phage ORFans may be partially due to the expected vast viral diversity, and may be instrumental in understanding viral evolution. The functions, origins and fates of the majority of viral ORFans remain a mystery. Further computational and experimental studies are likely to shed light on the mechanisms that have given rise to so many bacterial and viral ORFans.

## Background

Genome-wide studies of microbial ORFans have been carried out for about ten years now [1,2]. One of the major surprises in these studies is the large percentage of ORFs (Open Reading Frames) having few or no homologs in the databases [3,4]. These are referred to as ORFans [1]. With hundreds of complete prokaryote genome sequences, it has become evident that the presence of many ORFan genes is a natural phenomenon [3-7], that will continue to be observed for years. Recent studies have suggested that most ORFans are likely to correspond to real, expressed and functional proteins [8-13]. Despite studies focusing on particular bacterial lineages [14], the origin and functions of ORFans remain a mystery [6,15,16]: If proteins in different organisms have descended from common ancestral proteins by duplication and adaptive variation, why is it that so many today show no similarity to each other [4,15]? Why is it that we do not find today any of the necessary "intermediate sequences" that must have given rise to these ORFans? Do most ORFans correspond to rapidly diverging proteins [17,18]? If so, do they mainly correspond to nonessential proteins or to the species determinants?

Regardless of their origin, ORFans may be of two types [4]. Some ORFans may correspond to proteins with unique functions not currently observed in other families. Alternatively, ORFans may correspond to rapidly evolving and highly divergent members of known protein families, but with functions similar to proteins already known. Notice that our ORFan definition does not depend on database annotations beyond the mere ORF identification or on assumptions about the origin of ORFans. We use a merely operational definition: an ORFan is simply an ORF with no detectable sequence similarity to other ORFs in the database considered. This definition allows us to objectively identify and quantify ORFans, a required first step towards attempting to understand their functions and origins.

Recently, there has been a renewed interest in viral genomics [19], in part because of the realization of the major role that viruses, and in particular phages, have played in evolution. So far, the complete genome sequences of more than a thousand viruses, including hundreds of phages, have been deposited in the public database. Recent studies of phage genomes have revealed that horizontal transfer (HT) has played a major role in viral genome evolution [20,21]. Phages exchange genes with other phages mostly when they are inside the same host cell and with prophages residing in the host genome [22,23]. Phages can also exchange genes with their hosts, by integrating them as prophages or by exchanging individual genes with their hosts via recombination [24-26]. In particular, recent sequencing and comparative analyses of cyanophages and cyanobacteria has revealed some cases of HTs from hosts to phages [27-29].

Here we extend our ORFan studies to the viral world by addressing the following questions: 1) what is the percentage of ORFans in viruses and phages, how does it compare to the percentage of ORFans in bacteria, and how is this percentage related to the current phage sampling and to phage diversity? 2) Do viral ORFans show particular characteristics regarding length and GC content, as bacterial ORFans do? 3) How scarce is the current viral sampling, and are current observations likely to hold after many more genomes are sequenced? 4) What role do phage ORFans play in horizontal transfer from and to bacterial hosts?

## Results and discussion

The viral sequence data used in this work was obtained by downloading all 1,456 virus genomes available at Refseq [30] on September 2005. These genomes encode a total of 43,566 ORFs. We refer to this set of ORFs as our "All-Virus-DB". Out of the 1,456 virus genomes, 280 are phages, encoding a total of 18,368 ORFs. We refer to this set of phage ORFs as our "Phage-DB".

### ORFans collection

A viral ORF is defined to be a viral ORFan if a BLASTP search against our All-Virus-DB finds no significant hits outside its residing genome (see Material and Methods). 13,078 (30.0%) viral ORFs were thus identified as viral ORFans, a figure similar to that previously reported [31]. Notice that the percentage of viral ORFans is much larger than that of bacterial ORFans (9.1% in [6]). This confirms previous observations suggesting that the diversity among viruses is expected to be significantly larger than that among bacteria [31,32].

It is important to point out that our identification of ORFans is highly dependant on the quality of gene identification programs used to generate the list of ORFs in the database, especially the shorter ones. ORFs may be under-predicted (false negatives in gene prediction, and thus should be present in the database), or over-predicted (false positives in gene prediction, and thus should not be present in the database). It is beyond the scope of this paper to attempt to improve the ORF identification programs or to estimate the ratio of under- versus over- predicted ORFs in vira. Nevertheless, to partially overcome the problem of over-prediction of short ORFs that may not correspond to real genes, we have repeated all computations considering only ORFs longer than 300 bp (see Dataset Controls in Methods below). Previous works have shown that it is more unlikely for a longer ORF to be a false positive [3,33]. We believe that using this length threshold, we have significantly reduced the number of possible short false positives without removing a significant number of true-positive short ORFs.

### Special characteristics of ORFans

**Figure 1:**
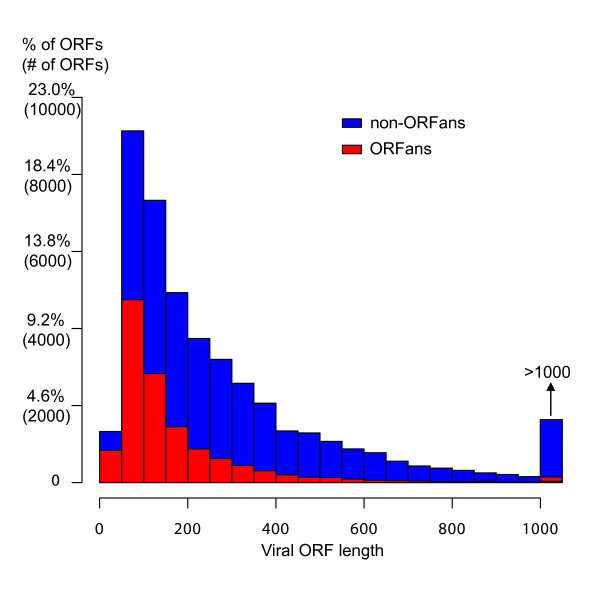
Viral ORFans are shorter than non-ORFans on average. The mean ORF length of ORFans is 172, whereas that of non-ORFans is 356. The histogram shows the length distribution of all viral ORFs. The lower bars correspond to viral ORFans.

It has been observed that bacterial ORFans have a number of characteristics different than those found in bacterial non-ORFans [3,14,34]. One of these is length: bacterial ORFans are shorter than non-ORFans on average (mean value 159 vs. 327 residues; p-value < 2.2e-16). To test whether viral ORFans are also shorter than viral non-ORFans, we computed the length distribution of viral ORFans and non-ORFans (Figure 1). As is clear from the figure, viral ORFans are also shorter than non-ORFans, with mean length values similar to those of Bacteria (172 vs. 356 residues; p-value < 2.2e-16).

Another reported characteristic of bacterial ORFans is that they have significant lower GC content than non-ORFans [14,34]. The GC3 content (GC at the third codon positions [34]) of bacterial ORFans is 51.4% whereas that of bacterial non-ORFans is 54.2% (p-value < 2.2e-16). To test whether this also holds for viruses, we computed the average GC3 content of viral ORFans and compared it to that of viral non-ORFans. The mean GC3 content of viral ORFans is 44.7%, also significantly lower than that of viral non-ORFans (45.9%; p-value = 7.0e-13). Nevertheless, notice that the difference in GC3 content between ORFans and non-ORFans is smaller and statistically less significant in viruses than it is in bacteria.

**Figure 2:**
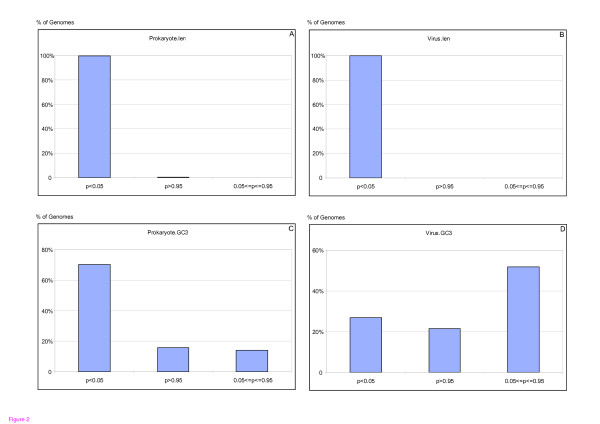
Length and GC3 content of ORFans and non-ORFans. While ORFans are statistically significantly shorter than non-ORFans in the majority of genomes, they have statistically significantly lower GC3 content than non-ORFans in a smaller fraction of the genomes. We applied the Wilcoxon nonparametric test to individual genomes. The null hypotheses were that ORFans are not shorter than non-ORFans, and that they do not have lower GC3 content than non-ORFans. For each genome, we compared the protein length and GC3 content distributions of its ORFans and non-ORFans. A p-value < 0.05 rejects the null hypothesis, and significantly supports the alternative hypothesis. A p-value > 0.95 supports the null hypothesis and a p-value between the two values means no significant difference. Only genomes with at least 30 ORFans and 30 non-ORFans were considered. The upper panels show the results of the length test on prokaryotes (left) and viruses (right). The lower panels show the results of the GC3 content test on prokaryotes (left) and viruses (right). Clearly, while the lower GC3 content of ORFans is statistically significant for about 70% of the bacterial genomes, it is statistically significant only for 27% of the viral genomes.

However, it is important to point out that using averages can be dangerous, as averages do not take into account the properties of individual genomes. While the observation that ORFans are shorter on average than non-ORFans holds for the vast majority of individual genomes, the observation that ORFans have lower GC content does not. The percentages of individual genomes that show that ORFans are statistically significantly shorter than non-ORFans are 99.6% and 100%, among bacterial and viral genomes, respectively (Figure 2A–B). But the percentages of individual genomes that show that ORFans have statistically significant lower GC content than non-ORFans are only 70.2% and 26.8% (!) among the bacterial and viral genomes, respectively (Figure 2C–D).

### Focusing on phages: higher percentage of ORFans in phages

**Figure 3:**
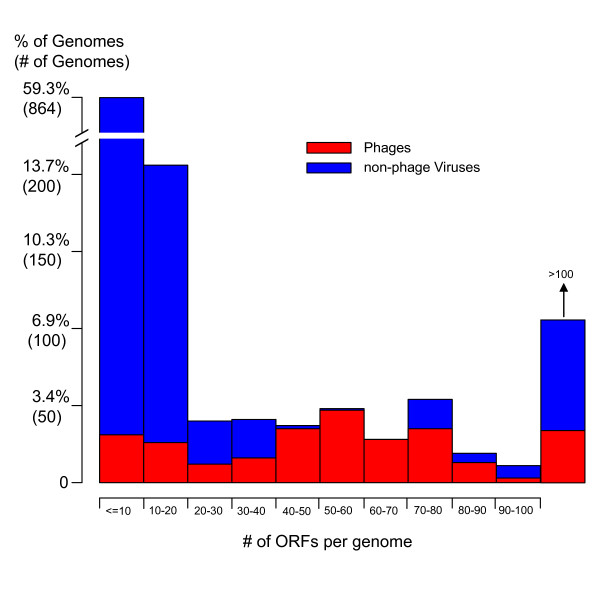
Distribution of the number of ORFs per genome in 1,456 viruses. Phages have a less extreme distribution of number of ORFs per genome than that of the non-phage viruses.

The 1,456 genomes in our All-Virus-DB comprise a very diverse set of organisms as it includes a variety of distantly related viral species with very diverse lifestyles: the 3 most abundant virus classes are ssRNA positive-strand viruses with no DNA stage (450 genomes), dsDNA viruses with no RNA stage (425 genomes), and ssDNA viruses (244 genomes). In addition, the viruses in our All-virus-DB show a great variability in genome size. Figure 3 shows the distribution of genome size (measured as the number of ORFs per viral genome) in our All-virus-DB. The average number of ORFs per genome is 30. Most of the viruses have very small genomes: 73.3% of the viruses have genomes with fewer than 20 ORFs (85 genomes have only one ORF), while only 7.4% of the viruses have more than 100 ORFs (the 3 largest genomes are: *Acanthamoeba polyphaga mimivirus*, *Paramecium bursaria Chlorella virus *and *Shrimp white spot syndrome virus *with 911, 690 and 531 ORFs, respectively).

The red bars in Figure 3 correspond to those viruses identified as phages (280 in total). Compared with the other viruses, phages have less extreme distribution of number of ORFs per genome: 163 out of the 280 (~60%) phages have between 40 and 100 ORFs in their genome. In addition, phages are mainly restricted to one taxonomical class: 75% of phages (211) correspond to dsDNA viruses with no RNA stage. In what follows, we study these 280 phages in more detail. We refer to the set of 280 phages as our "Phage-DB", which contains a total of 18,368 ORFs. Notice that phages correspond to less than 20% (280/1456) of all viral genomes, while the total number of phage ORFs correspond to more than 42% (18,368/43,566) of all viral ORFs.

Out of 18,368 phage ORFs, 7,047 (38.4%) correspond to phage ORFans (no homologs in other phages), significantly larger than the percentage of ORFans among all viruses (30.0%). This suggests that the diversity among phages is larger than that among all viruses. Interestingly, all but 282 (4.0%) out of the 7,047 phage ORFans correspond to viral ORFans too (no homologs in All-Virus-DB).

### Growing number of ORFans

**Figure 4:**
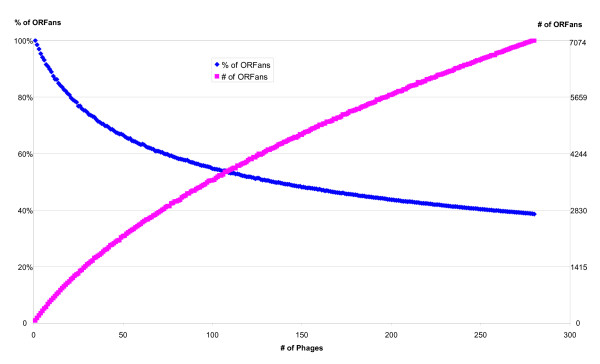
The number of phage ORFans is growing. The plot was computed by averaging the results of 1000 random selections of subsets of the 280 phage genomes. The average number of ORFans in each subset size is shown in red, and the percentage of ORFans (computed as the number of ORFans divided by the total number of ORFs) is shown in blue.

Figure 4 shows the growth in the number of ORFans and the decrease in the percentage of ORFans as a function of the number of available phage genomes. The figure suggests that although the percentage of ORFans is gradually decreasing, it is not likely to drop significantly even after hundreds of more phage genomes are sequenced. It also suggests that the actual number of ORFans will continue to grow as more genomes are sequenced. These trends in phages are consistent with those observed in prokaryotes [4,5].

To estimate the dependence of the number of ORFans to our database of fully sequenced viruses, we have searched for homologs of our identified viral ORFans within the recent (as of May 22, 2007) nr [35] and env_nr [36] (which includes the 6 million recently published predicted marine metagenomic proteins [37]) databases. We found that out of our 13,078 identified viral ORFans, 4878 have homologs in these databases. This demonstrates that the abundance of current ORFans is partially due to the number of fully sequenced organisms, and that a fraction of current ORFans will become non-ORFans as more complete genomes are sequenced. However, as we have previously demonstrated [3], the number of new ORFans in newly sequenced genomes is greater than the number of older ORFans that become non-ORFans. Indeed, it is estimated that >91% marine viral genes are novel [38]. Consequently, as more complete genomes are sequenced, the total number of ORFans will continue to grow, and our figure of 30.0% viral ORFans is not likely to vary dramatically in the coming years [4].

### ORFans in different phage groups

It has been estimated that each microbial species is a host for at least 10 phage species, suggesting the phage diversity is at least 10 times higher than microbial diversity, comprising about 10^8 ^species [39]. Nevertheless, there are only 280 phage genomes in our Phage-DB, suggesting that at least to some extent, the very high percentage of phage ORFans is a result of low phage sampling. To further explore this possibility, we studied the ORFans percentages in different phage groups.

Because phages with phylogenetically close hosts are often similar to each other [22,40-44], we classified the 280 phages according to their host ranges. We identified the hosts attacked by these phages, by automatically parsing the corresponding Genbank format files, or manually checking the related literature and searching against NCBI taxonomy database (Additional file 1). In Table 1, we list the taxonomical groups of the hosts identified, showing the number of sequenced prokaryotes, the number of sequenced phages, the sampling ratio and the percentage of phage ORFans. The table lists the 12 major lower level groups (those with less than 5 phages are grouped as "Others"), and the three higher level groups: Firmicutes, Gamma-Proteobacteria, and "non-Firm-Gamma". The average phage sampling ratio in each group is computed as the number of prokaryotes divided by the number of phages. This ratio reflects the current sampling status for each phage group. A sampling ratio of 10 would suggest that microbes are infected by 10 phages on average. However, the overall average ratio for all phages is only slightly higher than 1.0 (280 phages/277 prokaryotes), clearly showing that the current phage sampling is very low; the highest ratio is 3.3 for the Sulfolobales group, and the lowest (excluding "Others") is 0.38 for the Cyanobacteria group. The table also shows that the current sampling is biased towards Firmicutes and Gamma-Proteobacteria, the two most prevalently studied bacteria phyla [6]. In addition, within each phage group, the sampling is also biased, often toward some intensively studied bacteria. For example, 43 out of 62 Enterobacteria phages infect *Escherichia coli*; 35 out of 48 Bacillales phages infect *Staphylococcus aureus *and 12 out of 18 Pseudomonadales phages infect *Pseudomonas aeruginosa *(Additional file 1).

**Table 1:** Prokaryotes/Phage groups classified by phage host ranges and the percentage of ORFans within each group

Prokaryotic/Phage group	# of prokaryotes	# of phages	Sampling ratio	% of Phage ORFans
Bacteria_Proteobacteria_Gammaproteobacteria_Enterobacteriales	24	62	2.58	33.0%
Bacteria_Firmicutes_Bacilli_Bacillales	27	48	1.78	26.1%
Bacteria_Firmicutes_Bacilli_Lactobacillales	20	44	2.20	31.9%
Bacteria_Actinobacteria_Actinobacteria_Actinobacteridae	19	20	1.05	56.6%
Bacteria_Proteobacteria_Gammaproteobacteria_Vibrionales	6	18	3.00	77.6%
Bacteria_Proteobacteria_Gammaproteobacteria_Pseudomonadales	9	18	2.00	88.7%
Bacteria_Proteobacteria_Betaproteobacteria_Burkholderiales	9	15	1.67	41.7%
Archaea_Crenarchaeota_Thermoprotei_Sulfolobales	3	10	3.33	53.2%
Bacteria_Firmicutes_Mollicutes	14	9	0.64	64.8%
Bacteria_Cyanobacteria	13	5	0.38	57.0%
Bacteria_Chlamydiae	10	5	0.50	6.8%
Others	123	26	0.21	

All Gamma	63	109	1.73	40.2%
All Firmicutes	66	102	1.55	27.1%
All non-Firm-Gamma	148	69	0.47	53.7%

Table 1 also shows that the percentage of ORFans varies significantly among the groups (last column). Firmicutes have the fewest proportion of ORFans (27.1%) and "non-Firm-Gamma" have the largest (53.7%), reflecting the fact that the lowest sampled group has the highest percentage of ORFans. We would expect to observe a large negative correlation between the phage sampling ratios and the percentage of ORFans, if the following three conditions held: 1) the high percentage of ORFans is mainly influenced by the sampling; 2) different phage groups have similar diversities and 3) the phages that infect prokaryotes are evenly distributed. However, we do not observe any correlation between the phage sampling ratios and the percentage of ORFans (Spearman's rank correlation rho = -0.17, p-value = 0.61). This may be mainly due to the biased phage sampling and the varying diversities among the groups. For example, the Pseudomonadales phages contain the highest percentage of ORFans (88.7%), despite having a relatively large sampling ratio (18 phages for 9 bacteria). This suggests a relatively high phage diversity in this group [42]. In contrast, the Chlamydiae phages contain the lowest percentage of ORFans (6.8%), despite having a relatively low sampling ratio (5 phages for 10 bacteria). Interestingly, these 5 phages are all ssDNA viruses, whose diversity was not thought to be as high as that of dsDNA phages [43].

In summary, the above analysis shows that the current phage sampling is biased and scarce, corroborating previous observations [6,39]. Thus, any conclusions derived from the current data should be taken as preliminary observations only. Nevertheless, we believe that the data today already allows us to begin genome-wide studies, and some major trends already observed may be highly informative and may hold, at least qualitatively, when many more phages are sequenced.

### ORF conservation in the viral and prokaryotic world

**Figure 5:**
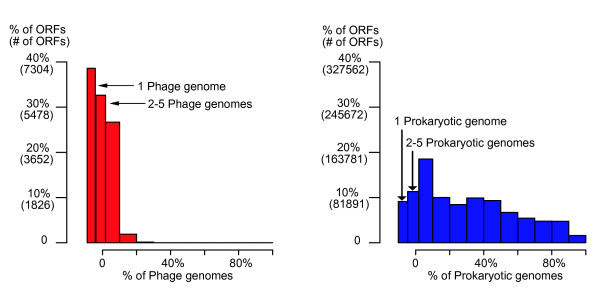
Histograms of H-value percentages for all phage ORFs (left) and all prokaryotic ORFs (right). The width of bins showing ORFans (1 phage/prokaryotic genome) and near-ORFans (2–5 phage/prokaryotic genomes) are expanded. Comparison of the two distributions clearly shows that there is a much lower conservation among phages than in prokaryotes.

We investigate the degree of conservation of phage ORFs, measured as the number of detectable phage homologs per ORF. For each ORF o, we compute its H value, defined as the number of phage genomes that contain homologs to o [6]. Notice that ORFs with H = 1 correspond to ORFans, and low values of H correspond to narrowly distributed ORFs and near ORFans. Figure 5 (left panel) shows the histogram of H-value percentages (H value divided by the total number of phages: 280) for all phage ORFs.

The figure shows that the phage ORFeome is highly diverse: in addition to the large percentage of ORFans (38.4%; H = 1), about a third (32.4%, 5,959 ORFs) of all phage ORFs have homologs in less than 5 phages (2< = H< = 5), and no phage ORF is conserved in more than 30% (H>84) of all the phages. As a comparison, the right panel shows the corresponding H-value percentage plot for prokaryotic ORFs, computed using a database of 277 prokaryotic genomes containing a total of 820,768 ORFs [6]. The figure shows that the prokaryotic ORFeome is less diverse than the phage ORFeome: the percentage of ORFans and near-ORFans are much lower than in phages (9.1% ORFans [H = 1] and 11.3% near ORFans [2< = H< = 5]) and 42.6% of bacterial ORF are conserved in at least 30% of the genomes.

### HT between viral and prokaryotic worlds

**Figure 6:**
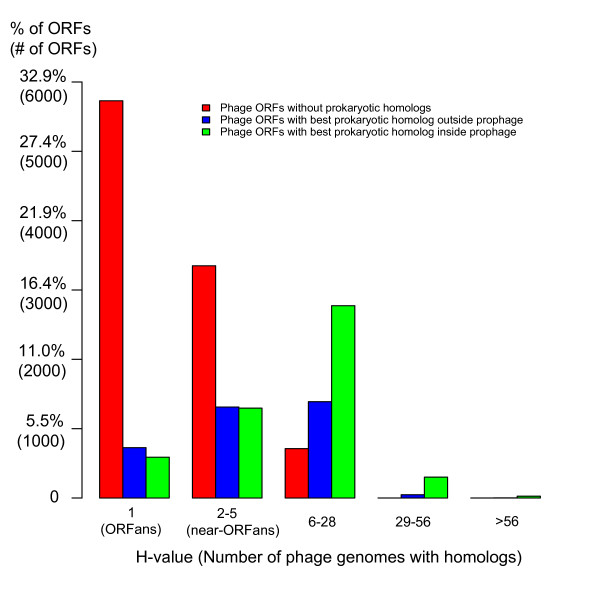
Bar plot of the H-value percentages of phage ORFs. On the basis of the left panel of Fig. 5, three types of phage ORFs are shown. Red bins correspond to phage ORFs with no prokaryotic homologs. The other bins correspond to phage ORFs having homologs in prokaryotes, which include two types: those with their best homolog not in prophages (blue) and those with their best homolog within prophages (green). The less conserved a phage ORF is within phages, the less likely it is to be a prokaryotic homolog. In addition, the more conserved a phage ORFs that has a prokaryotic homolog is, the more likely it is that its best homolog resides as a prophage.

It has been observed that there is a large phage gene pool residing inside prokaryotes (as prophages or phage-derived dispersed genes), suggesting extensive horizontal transfer (HTs) from phages to the hosts (phage-to-host HTs) [25,26]. In addition, HTs of host genes into phages (host-to-phage HTs) have also been observed [29,45]. Therefore, we should expect to find prokaryotic homologs for those phage ORFans and non-ORFans involved in HT. That is, some of the phage ORFans are not absolute ORFans when considering the prokaryotic ORFeome. To attempt to identify and quantify HTs between the two worlds, we carried out BLAST searches for each phage ORF (ORFans and non-ORFans) within the 280 phages in our Phage-DB against our database of 277 complete prokaryotic genomes. Figure 6 depicts our results as a function of the phage ORF H-value computed above.

Considering phage ORFans only, we found prokaryotic homologs for only 18.7% of the phage ORFans (1,317 out of 7,047, blue and green bars in the first column of Figure 6). Interestingly, the corresponding computation in prokaryotes showed that only 1.7% of the bacterial ORFans have homologs in the viral world [6]. By focusing on the best prokaryotic match, we find that 44.7% of these ORFans' best homologs (589 out of 1,317, green bars) correspond to prophages within a prokaryotic genome, clearly indicating that they correspond to phage-to-host HTs. In contrast, we found prokaryotic homologs for 63.8% of the phage non-ORFans (7,150 out of the 11,212 phage non-ORFans, blue and green bars in the other columns), and 61.5% of these non-ORFans' best prokaryotic homologs (4,397 out of 7,150) correspond to prophages. This shows that (i) the percentage of phage non-ORFans with prokaryotic homologs is significantly higher than that of the phage ORFans (63.8% vs. 18.7%) and that (ii) the more conserved a phage ORF is within the phage world (higher H-value), the more likely it is to have homologs in the prokaryotic world, and the more likely it is that its best homolog resides within a prophage; in particular, all of the phage ORFs (1,624) with H> = 15 have homologs in prokaryotes (80.4% of which reside in prophages). These findings are consistent with those recently observed in mycobacteriophages [44]. We propose that this may be a consequence of the simple fact that a better conserved phage ORF (with a larger H-value) means that our database contains more phages that can potentially transmit a homolog to prokaryotes. Considering phage ORFans and non-ORFans together, we find that 46.4% (8,467) phage ORFs have homologs in the prokaryotic world, of which 58.9% (4,987) have their best homologs within prophages. This suggests that over one fourth of the phage ORFs have been involved in HT from the phage to the bacterial world.

While determining HT from phage to bacteria on the basis of finding prophage homologs is relatively straightforward, other types of HT (host to phage HT or phage to host HT, but not as prophages) are more difficult to identify. While some studies have already identified cases of host to phage HT [27], further studies are required to estimate the overall fraction of phage ORFans and non-ORFans that correspond to horizontally transferred ORFs from bacteria. The fact that the percentage of ORFans with bacterial homologs is significantly lower than that of non-ORFans suggests that many phage ORFans may correspond to phage specific functions. Thus phage ORFans will likely turn out to be major players in the enormous phage diversity. Further sampling of bacterial and phage genomes will undoubtedly contribute to a better understanding of the evolution and the relationship between these two worlds.

## Conclusion

We have carried out a first systematic analysis of viral ORFans. We have found that the percentage of ORFans in the virus world is much higher than the percentage of ORFans among bacteria. We have found that, like in the bacterial world, viral ORFans are shorter than non-ORFans on average, and that this difference is statistically significant in the vast majority of individual genomes. We have also found that, like in the bacterial world, viral ORFans have a lower GC3 content than non-ORFans on average. However, when studying individual genomes we found that the difference is statistically significant only for a small percentage of the individual phage genomes. We found that while a majority of phage non-ORFans have bacterial homologs (61.5% of which are likely to be involved in phage to host horizontal transfer), only a small percentage of phage ORFans have bacterial homologs (and only a small fraction of which are involved in phage to host HT). Because the current sampling of phages (and of bacterial genomes in general), is limited and biased towards particular groups, the percentage of ORFans in different phage groups varies significantly. This low sampling may be a factor contributing to the abundance of phage ORFans, but is not likely to be the only one. That is, even after many more genomes are sequenced, we expect to find a significant number of ORFans and near-ORFans, awaiting interpretation. There are also other possibilities to account for the ORFans' origin, like rapid divergence after horizontal transfer (from hosts or from other viruses, from existent genomes or yet extinct genomes) or duplication. Future studies are required to elucidate how much these possibilities account the most for what viruses: e.g. marine phages have been thought to have grasped many genes from their hosts [27]. Unraveling the mystery of the origins and functions of ORFans will likely remain a major challenge.

## Methods

### Protein and genomic data

Virus proteins and genomic data in Refseq release 13 (Sept. 13, 2005) were downloaded from [46]. 43,566 viral proteins are cross referenced to 1,456 NCBI taxonomical species. Another 72 viral species not encoding proteins in the Refseq release 13 were excluded in our analyses. 277 fully sequenced microbial proteomes and genomes (Nov. 03, 2005) were downloaded from [47], as described in [6].

### ORFan identification

Viral ORFans were identified using a procedure similar to the one we have used in previous work [6]: A viral ORF is defined to be a viral ORFan if a BLASTP search against our All-Virus-DB finds no significant hits outside its residing genome. We define a significant hit if the BLASTP e-value is lower than 1e-3 (or for alignment lengths < 80aa, 1e-5).

### Prophage identification

We identified prophages residing in the prokaryotic genomes using our own perl scripts implementing a method similar to that used by Bose and Barber [48].

### Dataset controls

To investigate the possible dependence of our results to the particular data used, and to attempt to quantify the extent of any present bias, we have carried out three control experiments. In each control, we applied the exact same computations as the ones described in the text, and verified that the results remain statistically significant. The three control experiments exclude subsets of the data as follows: a) Excluding shorter ORFs (< = 300 bp) b) Excluding smaller genomes (<50 ORFs) and c) Excluding ssDNA and all RNA viruses. In all three cases, our main observations remain statistically significant. A detailed summary of each of these controlled can be found at [49].

## Authors' contributions

YY and DF designed the research. YY conducted the computation, analyzed data, and drafted the manuscript. DF supervised this project and finalized the manuscript. All authors read and approved the final manuscript.

## Supplementary Material

Additional File 1 280 fully sequenced phages and their hosts. Taxonomy information and corresponding bacterial host for each phage. Click here for file
